# Effects of *Laetiporus sulphureus*-Fermented Wheat Bran on Growth Performance, Intestinal Microbiota and Digesta Characteristics in Broiler Chickens

**DOI:** 10.3390/ani10091457

**Published:** 2020-08-20

**Authors:** Wei Chih Lin, Tzu Tai Lee

**Affiliations:** 1 Department of Animal Science, National Chung Hsing University, Taichung 402, Taiwan; waynezi22@smail.nchu.edu.tw; 2 The iEGG and Animal Biotechnology Center, National Chung Hsing University, Taichung 402, Taiwan

**Keywords:** *Laetiporus sulphureus*, intestinal microbiota, metagenomics, broilers

## Abstract

**Simple Summary:**

This study investigated the effects of a *Laetiporus* *sulphureus*-fermented wheat bran (LS) supplementation on the microbiota and digesta characteristics of broiler chickens. Results showed that a 5% LS supplementation could potentially enhance the feed conversion ratio and European Broiler Index (EBI) of the broilers by elevating the family Lactobacillaceae and suppressing the phylum Proteobacteria’s population. This could also change the intestinal environments by elevating the ileal and cecal lactic acid concentrations as well as lowering the pH and ammonium nitrate, thereby potentially favoring the growth and health of the broilers.

**Abstract:**

This study investigated the effects of a *Laetiporus sulphureus*-fermented wheat bran (LS) supplementation on the microbiota and digesta characteristics of broiler chickens. Two hundred and forty male broilers (Ross 308) were randomly allocated into three groups fed with a corn–soybean-based diet (control), and the control diet being replaced with 5% wheat bran (WB) and 5% LS, respectively. Each group had four replicates and 20 birds per pen. Metagenomics analysis results of the ileum microbiota showed that, at the family level, the 5% LS groups had over 40% higher Lactobacillaceae compared to the control group in a mean difference comparison. Heat maps showed that, at the phylum level, the population of Firmicutes was higher and Proteobacteria was lower in the ileum of 5% LS compared to the control group. Results of the stack column plots of the top ten OTUs at the family level showed that a 5% LS and 5% WB supplementation altered the broiler microbiota distribution by increasing the relative abundance of Lactobacillaceae. Cecal microbiota analysis showed that the 5% LS-supplemented group had approximately 5% and 3% higher Veillonellaceae and Lactobacillaceae, respectively. Stack column plots of the top ten OTUs indicated that the distribution of cecal bacteria in each group was not markedly different. Both the ileum and cecum digesta in the 5% LS supplementation group had a slight and not significant elevation on the total VFA, while the pH values and ammonia nitrogen were significantly lowered compared to the control and 5% WB groups (*p* < 0.05). In addition, the 5% LS supplementation group had a significantly higher lactic acid concentration in both the ileum and cecum compared to the control and 5% WB groups (*p* < 0.05). In conclusion, a 5% LS supplementation could potentially enhance the feed conversion ratio and European Broiler Index (EBI) of broilers by elevating the family Lactobacillaceae and suppressing the phylum Proteobacteria’s population, thus creating changed intestinal environments that may potentially favor the growth and health of the broilers.

## 1. Introduction

Intestinal microbiota is a pivotal composition in broilers as it plays a crucial role in modulating physical functions, such as nutrition, immunity, and metabolism. These functions also modulate the intestinal integrity, oxidative status, and inflammatory status, which are eventually linked to factors such as energy balance, feed efficiency, and growth rate, as they decide growth performance [1]. The modification effects of intestinal microorganisms on the use of dietary metabolizable energy in broilers have been reported in research [2]. During unfavored conditions, the transformation of gastrointestinal tract (GIT) microbiota can impair the intestinal morphology and induce an inflammation process, which could exploit the energy expenses and growth of the chickens and eventually suppress growth performance [3]. Therefore, the balance of intestinal microbiota is important to promote a healthy gut and the maximum growth performance of chickens [4].

Feed composition is a major factor that modulates the GIT bacteria and may affect the gut health of animals either positively or negatively, depending on the type of diets [5]. Wheat bran (WB) has been reported as a potential alternative material for major crop feedstuffs as it could save the rising feed costs caused by the increasing demand for grains [6]. Over 6.5 million tons of wheat are produced globally per year, and WB is an agricultural by-product got from wheat during the flour-making process. However, a high fibrous content, including the non-starch polysaccharides (NSPs) (44.0%) and low nutritional value (approximately 1300 kcal/kg of metabolizable energy), make WB challenging to be supplied to chickens [7]. It was reported that high concentrations of insoluble NSPs could shorten the dwelling time of digesta in the GIT, leading to insufficient time for feed digestion and nutrient absorption. Furthermore, soluble NSPs act as anti-nutritive substances in the body, which facilitate the fermentation rate in the intestine and act as source of energy for anaerobic microbes [8,9]. They help to propagate harmful pathogen like *Clostridium perfringens*, which causes various diseases in poultry [8,9]. In addition, WB was reported to have anti-nutritional effects that could inhibit digestibility, causing pathogen proliferation in the gastrointestinal tract and inducing gut inflammation due to the high content of NSPs [10]. It has been reported that filamentous fungus fermentation on WB could eliminate the negative effects of NSPs by secreting NSP-degrading enzymes that promote the digestibility in broilers [6,10]. Moreover, the beneficial compounds produced, such as the phenolic compounds and polysaccharides, might also alter the composition of the GIT microbiota in broilers [6,11].

Fungi has been considered as one of the probiotics that promotes the health and productivity of animals. Shamsi et al. [12] showed that mushroom powder and flavophospholipol increased the yield of the carcass parts, such as breast and drumsticks. Furthermore, a multi-enzyme produced by a single fungus supplementation in a corn–soybean meal was shown to potentially improve the growth performance of broilers fed with a meal that had almost a 3% reduction in nutrients and housed in a density of 16 birds/m^2^ compared with the negative control [13]. Moreover, fungal fermentation was proven to increase the nutritional value of some low-value feedstuffs. *Pleurotus florida* fermentation was demonstrated to improve the nutrition value and rumen degradability of wheat and barley straw [14]. Chu et al. [11] reduced the neutral detergent fiber (NDF) and acid detergent fiber (ADF) contents of WB by the solid-state fermentation (SSF) of *Trichoderma* spp. and successfully replaced the corn by 10% in the basal diet without negatively influencing the performances of the broiler chickens. Similarly, a *Trichoderma* spp. SSF also improved the NDF and ADF digestibility of rice straw in Barbados sheep compared to the unfermented rice straw [15]. Regarding to the modulation effects of a fungus, *Antrodia cinnamomea* was reported to reduce obesity via modulation of gut microbiota in mice fed high-fat diets [16]. *Ganoderma lucidum* polysaccharides was shown to have therapeutic effects on chronic pancreatitis by altering the composition of the intestinal microbiota [17]. Furthermore, Adams et al. [18] demonstrated that *Pleurotus ostreatus* mushroom decreased the incidence of diarrhea while increased the growth performance of weaned piglets by increased the diversity and richness of the microbiota community in the feces. However, despite the fact that various fungal species had been investigated with their versatile functions, the modulating effects on the intestinal microbiota of *Laetiporus* sp. has been little studied.

*Laetiporus* sp. is one of the medical fungi used traditionally by Europeans to cure pyretic diseases, coughs, gastric cancer, and rheumatism [19]. Recently, our study had shown that *L. sulphureus*-fermented WB (LS) can potentially enhance growth performance and improve intestinal microflora and inflammation status in broilers. The study tested limited indexes to evaluate the effects of LS on the intestinal microbial composition, such as coliforms and lactic acid bacteria, by applying the traditional plate count method [20]. However, the in-depth understanding of the effects of *L. sulphureus* on intestinal microbiota in broilers and its relativities on growth performance required further investigation. Furthermore, considering that changes in the GIT microbiota could lead to the transformation of the intestinal environment, for instance, an increase in the *Lactobacillus* population might acidify the GIT by producing lactic acid and inhibiting the colonization of pathogenic bacteria [21], it is necessary to examine several indexes in intestinal digesta, such as the pH value, lactic acid content, ammonia nitrogen, and total volatile fatty acid (VFA). Therefore, this study used the next-generation sequencing (NGS) technique to determine the microbiota dynamics in the intestine, with increased coverage and accuracy, along with the indexes in the intestine for elucidating the effects of LS on manipulating the intestinal microbiota composition and intestinal environments in broilers.

## 2. Materials and Methods

### 2.1. Microorganism and Culture Method

The *Laetiporus sulphureus* (Bull.) Murril (BCRC 35305) used for this study was purchased from the Bioresource Collection and Research Center (BCRC), Food Industry Research and Development Institute (Hsinchu, Taiwan). The microorganisms were routinely maintained on a malt extract agar (MEA, malt extract 2%, glucose 2%, peptone 1%, and agar 2%) plate at 25 °C with regular sub-cultivation (no longer than 1 week).

### 2.2. Inoculum Preparation and Solid-State Fermentation (SSF)

Inoculum for the SSF was prepared by shake flask culture with malt extract broth (MEB). Briefly, 250 mL Erlenmeyer flasks filled with 100 mL of MEB were autoclaved for 30 min at 121 ± 1 °C. *L. sulphureus* was then transferred to the medium from the MEA plates; 5 pieces of agar were used for the inoculation of 100 mL of liquid media. Flasks with agar pieces in MEB were incubated at 25 °C on a rotary shaker incubator (PHCbi, Tokyo, Japan) at 120 rpm for 5 d. Before inoculation, MEB grown with *L. sulphureus* was put in a sterilized plastic bag and homogenized with Seward Stomacher (Seward Laboratory Systems Inc., Bohemia, NY, USA).

Solid-state fermentation of LS was performed in a heat-resistant plastic bag containing 50 g of WB, adjusted to 50% moisture with distilled water and thoroughly mixed and autoclaved at 121 ± 1 °C for 30 min. Subsequently, the autoclaved wheat bran was inoculated with 10 mL of homogenized inoculum and fermented aerobically under environmentally controlled conditions and maintained at 25 °C for 12 d. Samples were than dried at 40 °C for 2 d and ground in a mill before being supplemented in the broiler feeds.

### 2.3. Experimental Birds and Management

The procedure and animals used in this experiment were approved by the Animal Care and Use Committee of National Chung Hsing University, Taiwan (IACUC No. 105-140). A total of 240 1-day-old commercial Ross 308 male broiler chicks were randomly allocated to one of three groups: control (corn–soybean meal), 5% corn replaced by 5% WB (5% WB), and 5% corn replaced by 5% LS (5% LS), with 4 replicates and 20 birds per pen (total of 80 birds/treatment). Ambient temperature was maintained at 34 ± 1 °C for the first 7 d, and then gradually decreased to 26 ± 1 °C until the birds reached 21 d of age. After 21 d, the temperature was maintained at 26 °C until the end of the trial (35 d). The experimental period included 2 phases: a starter phase (1–21 d) and finisher phase (22–35 d). At the beginning of the feeding trial, the average body weights of the birds were even for all pens (approximately 43.4 g). The birds were kept in floor pens (2.5 × 4.0 m) with wire floors and rice hull as litter material. In the starter phase, two fountain drinkers and one feed tray were included per pen. Nipple drinkers and a feeder were used as replacement in the finisher phase. Vaccines for Newcastle disease and infectious bronchitis were provided to chicks immediately after birth. Water and feed were supplied ad libitum to the broilers. The feed formula (Table S1) was designed to meet the nutrient requirements of the broilers in line with the requirements of the NRC [22]. Starter and finisher diets were offered to the birds from 1–21 d and from 22–35 d of age, respectively. Neither anti-coccidial nor anti-bacterial supplements were added to the feed mixtures. On Day 35, the performance of the broilers was assessed by recording the feed intake, body weight of the birds, and average daily weight gains (ADG). After the calculation of the viability percentage and FCR (feed/gain), the European Broiler Index (EBI) was applied for the evaluation of the growth performance of the broilers according to the following formula suggested by Marcu et al. [23]:

(1) $$\mathrm{EBI}=\frac{\mathrm{ADG}\;\left(g\right)\times \mathrm{viability}\;\%}{\mathrm{FCR}}\times 10$$

### 2.4. DNA Sample Collection for Metagenomics Analysis

Cecal and ileal digesta in 35-day-old broilers were collected from 6 broilers of each of the control, 5% LS, and 5% WB groups. Samples were immediately isolated for their genomic DNA by using Quick-DNA Fecal/Soil Microbe Miniprep Kits (Zymo, Irvine, CA, USA); procedures were accomplished by the following manufacturer’s protocol. The extracted DNA qualities, including purity and concentration, were analyzed through a NanoDrop 2000 spectrophotometer (Thermo Scientific, Waltham, MA, USA).

### 2.5. Polymerase Chain Reaction and Sequencing

The 16S rRNA amplicons were measured and pooled for the sequencing reaction. The collection of 16S rRNA sequences were performed using a HiSeq 2500, PE250 (Illumina, Inc., San Diego, CA, USA). The DNA samples were amplified using the primer set that targets the V3–V4 region of bacterial 16S rDNA (319F: 5′-CCTACGGGNGGCWGCAG-3′/806R: 5′-GACTACHVGGGTATCTAATCC-3′). All polymerase chain reactions were conducted using the KAPA HiFi HotStart ReadyMix (Roche, Nutley, NJ, USA). Sequencing libraries were generated using the TruSeq^®^ DNA PCR-Free Sample Preparation Kit (Illumina, San Diego, CA, USA), as per the manufacturer’s recommendations, and the index codes were added. The library quality was assessed using a Qubit^®^ 2.0 Fluorometer (Thermo Scientific) and an Agilent Bioanalyzer 2100 system (Agilent, Santa Clara, CA, USA). Finally, the library was sequenced on an IlluminaHiSeq 2500 platform, and 300 bp paired-end reads were generated. After sequencing, whole tags were assembled using the UCHIME algorithm to detect chimera sequences; the chimera sequences were removed before the effective tags were obtained. Sequence analysis was performed using Uparse software (Drive5, Tiburon, CA, USA) (Uparse 135 v7.0.1001; http://drive5.com/uparse/). Sequences with ≥97% similarity were assigned to the same operational taxonomic units (OTUs). A representative sequence of each OTU was selected for further annotation. Alpha diversity was applied to analyze the complexity of species diversity for a sample by using six indices: Observed OTUs, Shannon, Simpson, Chao1, ACE, and PD whole tree. All the indices of our samples were calculated using Quantitative Insights Into Microbial Ecology (QIIME, v1.9.1). To evaluate differences in samples with respect to species complexity, a beta diversity analysis on a weighted unifrac was conducted by using Python software (Python Software Foundation, Wilmington, DE, USA) with QIIME protocol (v1.9.1). Principal co-ordinates analysis (PCoA) was performed at the genus level. LEfSe (linear discriminant analysis effect size) was performed to detect differential abundant taxa across groups using the default parameters.

### 2.6. Determination of Total Volatile Fatty Acids in Digesta

Total volatile fatty acid was determined by the method of Kromann et al. [24] with modification. Briefly, 5 g of digesta was added with 10 mL of distilled water and 10 mL of 1.5 M saturated MgSO_4_-sulfuric acid solution. The mixture was extracted under 4 °C overnight and centrifuged at 3000× *g* for 20 min. Five milliliter of the supernatant were added into a glass tube and distilled with the Kjeldahl method. The distillate was collected and titrated with 0.01 N NaOH; it was checked with a phenolphthalein indicator, and the percentage recovered was calculated as below:

$$\mathrm{VFA}\;\left(\mu \mathrm{mole}/g\right)\;=\;\frac{\mathrm{Titrated}\;\mathrm{NaOH}\;\mathrm{volume}\;\left(\mathrm{mL}\right)\times 0.01\times 3\times F}{5\;g\;\mathrm{sample}\;\mathrm{weight}}\times 1000$$

while F was the factor of NAOH.

### 2.7. Determination of the Ammonium-Nitrogen Concentration in Digesta

The ammonium-nitrogen concentration in the digesta was determined by the methods of Weatherburn [25] with modification. Briefly, 1 g of digesta was mixed and vortexed with a 4 mL 25% metaphosphoric acid solution. The mixture was than centrifuged for 10 min at 15,000× *g* under 4 °C, and 25 µL of the supernatant was than mixed with Reagent A (consisted of 0.05% phenol and 0.025% sodium nitroferricyanide) and Reagent B (consisted of 0.25% NaOH and sodium hydrochloride solution) and allowed to react at 37 °C in a water bath for 15 min. After the reaction time was finished, ammonia nitrogen was determined colormetrically at 630 nm, and calculated according to the standard curve using NH_4_Cl.

### 2.8. Determination of the Lactic Acid Concentration in the Intestinal Digest

The concentrations of lactic acid in the ileal and cecal digesta were analyzed using a high-performance liquid chromatography (HPLC) system (Hitachi, Tokyo, Japan). One gram digesta was added with 1 mL distilled water and 4 mL acetonitrite. It was filtered through a 0.22-μm membrane filter and subsequently analyzed using an HPLC instrument (HITACHI, Kyoto, Japan) equipped with a pump (L-2130), UV detector (L-2490), column (Gemini 5u C6-Phynel 110A 250 × 4.6 mm), and computer system with HPLC D-2000 Elite. The sample injection volume was 10 µL and the mobile phase was a 20 mM phosphate buffer with 3% methanol. Chromatographic peaks in the samples were identified by comparing their retention times and UV spectra (220 nm) with the reference standard (Lactic acid, Sigma-Aldrich, St. Louis, MO, USA). Working standard solutions (10 µL) were injected into the HPLC instrument to obtain the peak area responses. A standard curve and calibration formula for lactic acid was prepared by plotting concentration versus area. Quantification was conducted according to the integrated peak areas of the sample and the corresponding standard curves.

### 2.9. Statistical Analysis

Data were subjected to ANOVA as a completely randomized design using the GLM function of the SAS 9.4 (SAS Institute, Inc., Cary, NC, USA). Significant statistical differences among the various treatment groups means were determined using Tukey’s honestly significant difference test. The effects of the experimental diet on response variables were considered to be significant at *p* < 0.05.

## 3. Results

### 3.1. Growth Performance

The effects of LS and WB supplementation in the feed on the growth performance of 35-day-old broilers are displayed in Table 1. Results showed that the 5% LS had a significantly higher FCR and EBI compared to the 5% WB and control groups (*p* < 0.05). However, the FBW, ADG, and viability showed no difference across the groups.

### 3.2. Effects of LS Supplementation on Diversity of Ileal and Cecal Microbiota

**Figure 1:**
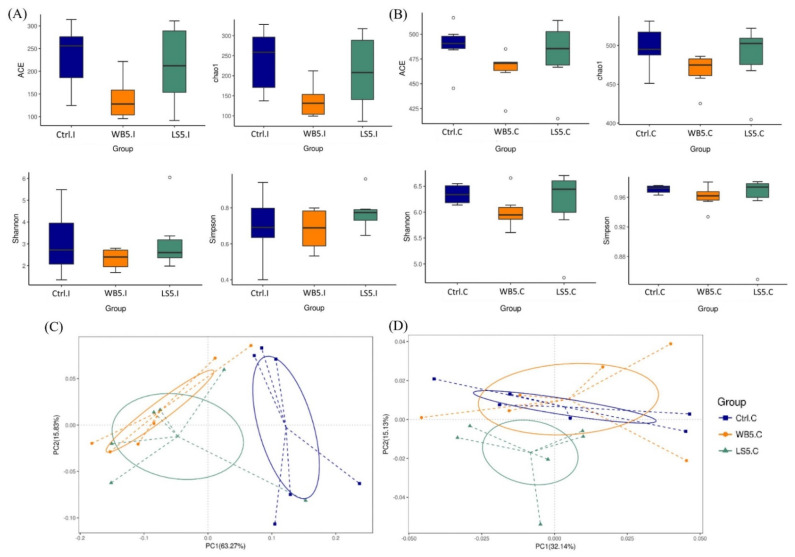
Diagram of alpha diversity in the ileum (**A**) and cecum (**B**), as well as the weighted PCoA showing beta diversity in the ileum (**C**) and cecum (**D**) of 35-day-old broilers. Ctrl: control group, WB5: broilers supplemented with 5% wheat bran, LS5: broilers supplemented with 5% *Laetiporus sulphureus* wheat bran. Results are means of six samples obtained from the individual birds of each of the control and experimental groups.

The alpha diversity of the ileal and cecal microbiota are shown in Figure 1A,B, respectively, and also Table 2. Results showed that there was no significant difference in the ACE, Chao1, Shannon, and Simpson diversity indices across the groups. Principal co-ordinates Analysis (PCoA) results showed that the ileal microbes in the control group were distinctively different from the 5% LS and 5% WB groups, while the 5% LS and 5% WB were similar. The principle components, PC1 and PC2, of the treatment groups were separated with 63.27% and 15.83% variation, respectively (Figure 1C). Results of the PCoA illustrated that the 5% LS group was partially separated from the 5% WB group, while the 5% WB and control groups were merely fully overlapped. The principle components, PC1 and PC2, of the treatment groups were separated with 15.13% and 32.14% variation, respectively (Figure 1D).

### 3.3. Heat Map of the Microbiota Composition in the Ileum and Cecum

**Figure 2:**
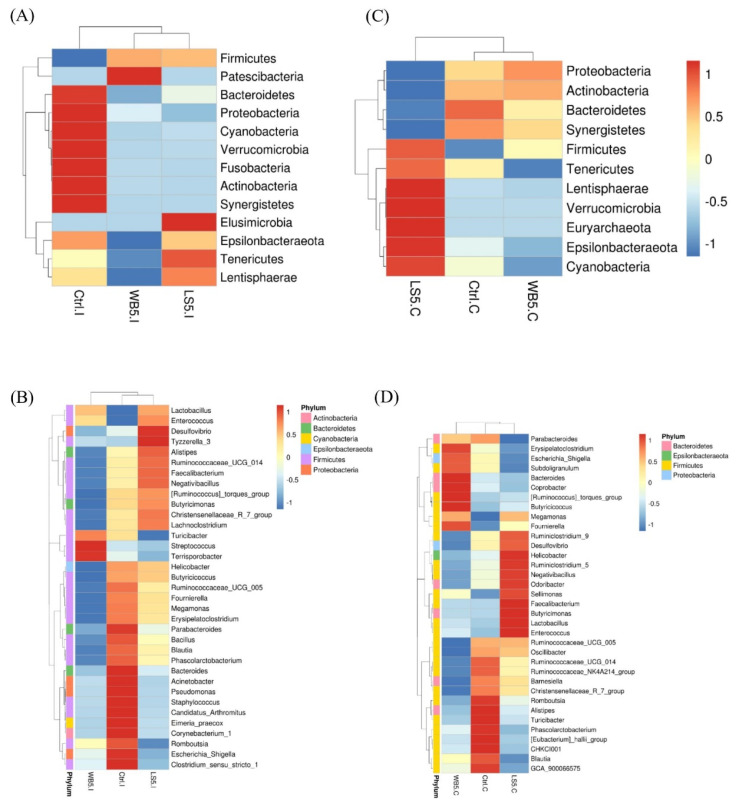
Heat maps showing the microbiota composition at the phylum and genus level in the ileum (**A**,**B**) and cecum (**C**,**D**) of 35-day-old broilers supplemented with WB and LS. Ctrl: control group, WB5: broilers supplemented with 5% wheat bran, LS5: broilers supplemented with 5% *Laetiporus sulphureus* wheat bran. Results are means of six samples obtained from the individual birds of each of the control and experimental groups.

The results of the microbiota structure showed by the heat map (Figure 2) indicated that, at the phylum level, Firmicutes in the ileum of the 5% LS and 5% WB groups were higher than in the control group, while Bacteroidetes, Proteobacteria, Cyanobacteria, Verrucomicrobia, Fusobacteria, Actinobacteria, and Synergistetes were higher in the control group. Elusimicrobia of the 5% LS group was higher than other groups, while the 5% WB-supplemented group had the lowest Epsilonbacteraeota, Tenericutes, and Lentisphaerae compared to other groups (Figure 2A). At the genus level, ileal *Lactobacillus* and *Enterococcus* of both the 5% LS and the 5% WB groups were higher than in the control group. The 5% LS group had higher *Desulfovibrio* and *Ruminococcaceae*, while the 5% WB had higher *Turicibacter*, *Streptococcus*, and *Terrisporobacter* than the 5% LS group and the control group (Figure 2B). The heat map showing the microbiota structure of each group indicated that when compared to the control and 5% WB groups, the 5% LS group consisted of more Firmicutes, Tenericutes, Lentisphaerae, Verrucomicrobia, Euryarchaeota, Epsilonbacteraeota, and Cyanobacteria at the phylum level (Figure 2C). At the genus level, the 5% LS group had more *Enterococcus*, *Lactobacillus*, *Butyricimonas*, *Faecalibacterium*, *Sellimonas*, *Odoribacter*, *Negativibacillus*, *Ruminoclostridum*, *Helicobacter*, and *Desulfovibrio* when compared to the control and 5% WB groups (Figure 2D).

### 3.4. Mean Differences in Microbiota Composition in the Ileum and Cecum

**Figure 3:**
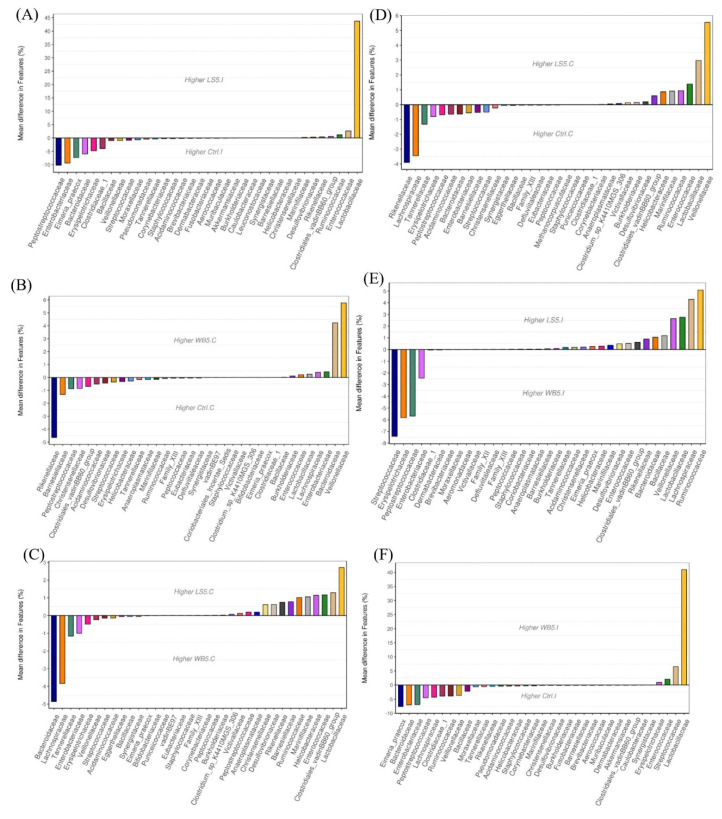
Mean differences in the microbiota composition at the genus level of 35-day-old broilers. Ileum of LS vs. Ctrl (**A**); WB vs. Ctrl (**B**); LS vs. WB (**C**); Cecum of LS vs. Ctrl (**D**); WB vs. Ctrl (**E**); LS vs. WB (**F**). Ctrl: control group; WB5: broilers supplemented with 5% wheat bran; LS5: broilers supplemented with 5% *Laetiporus sulphureus* wheat bran. Results are means of six samples obtained from the individual birds of each of the control and experimental groups.

A comparison of the mean differences at the family level in the ileum of the 5% LS group and the control group showed that the 5% LS group had over 40% higher Lactobacillaceae and the control group had approximately 10%, 9%, and 7% higher Peptosterptococcaceae, Entrobacteriaceae, and Eimeria praecox, respectively (Figure 3A). The 5% WB group, compared to the control group, had approximately 40% higher Lactobacillaceae and 6% higher Steptoccocaceae, while the control group had approximately 7.5% of *Eimeria praecox*, Bacteroidaceae, and Entrobacteriaceae. The 5% LS group, compared to the 5% WB group, had 5%, 4%, and 2% higher Ruminococcaceae, Lachnospiraceae, and Lactobacillaceae, respectively (Figure 3B). The 5% WB group, compared to the 5% LS group, had approximately 7.5% higher Streptococcaceae and 5.5% higher Erysipelotrichaceae, and also 2.5% higher Enterobacteriaceae (Figure 3C). The mean difference comparison in the cecum showed that, at the family level, the 5% LS group had approximately 5.5% and 3% more Vellionellaceae and Lactobacillaceae, respectively, compared to the control group, which had 3.5% and 4% more Rikenellaceae and Lachnosipraceae, respectively (Figure 3C). The 5% WB group had approximately 4% and 4.9% more Bacteroidaceae than the control and 5% LS groups, respectively (Figure 3D). The 5% LS group, compared to the 5% WB group, had approximately 2.5% more Lactobacillaceae (Figure 3E).

### 3.5. Top Ten OTUs of the Ileum and Cecum

**Figure 4:**
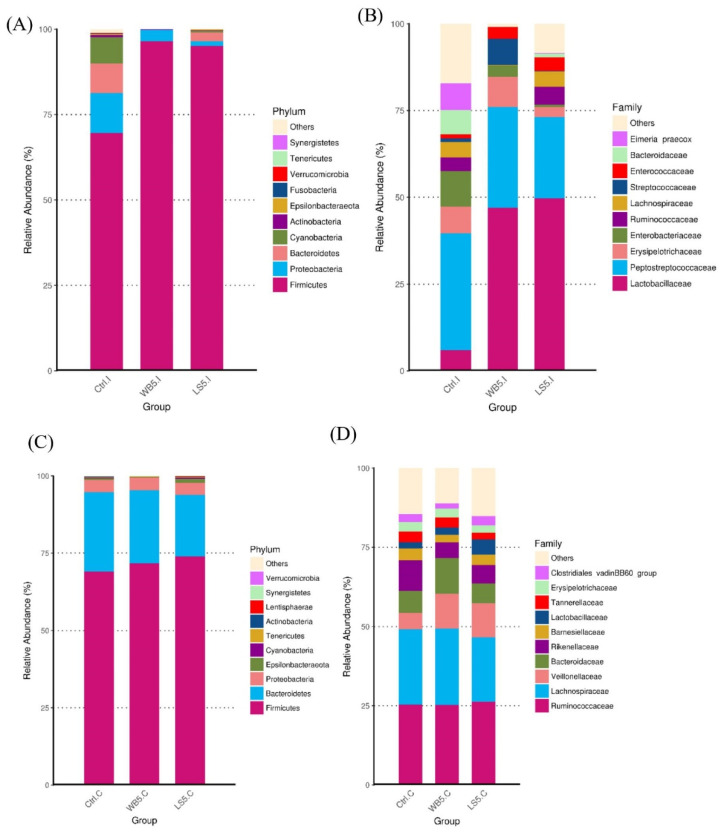
Top ten OTUs of the microbiota composition at the phylum and family level in the ileum (**A**,**B**) and cecum (**C**,**D**) of 35-day-old broilers. Ctrl: control group; WB5: broilers supplemented with 5% wheat bran; LS5: broilers supplemented with 5% *Laetiporus sulphureus* wheat bran. Results are means of six samples obtained from the individual birds of each of the control and experimental groups.

The change in the ileal microbiota composition was induced by both WB and LS supplementation. The relative abundance of the phylum Firmicutes, which dominated the ileum, was elevated to over 80% in both the 5% WB and 5% LS groups, while the control group had less than 75% Firmicutes (Figure 4A). At the levels of family, the relative abundance of the Lactobacillaceae of both the 5% WB and the 5% LS groups were significantly higher than the control group. Furthermore, it was obvious at the levels of family and genus that there was little relative abundance of the unclassified bacteria. The family Enterobacteriaceae and genus *Lactobacillus* was the lowest in the 5% LS group (Figure 4B). The cecal microbiota composition showed by stack plots of the top 10 OTUs at the phylum and family level showed similar patterns between each group (Figure 4C,D). However, it can be observed that, at the family level, unclassified bacteria were fewer than the control and 5% LS groups.

### 3.6. Linear Discriminant Analysis (LDA) Effect Size (LEfSE)

**Figure 5:**
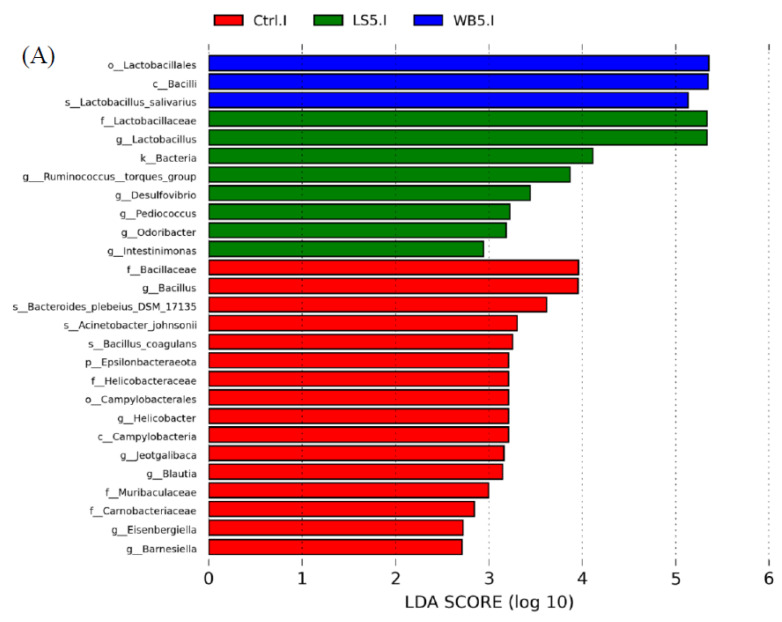

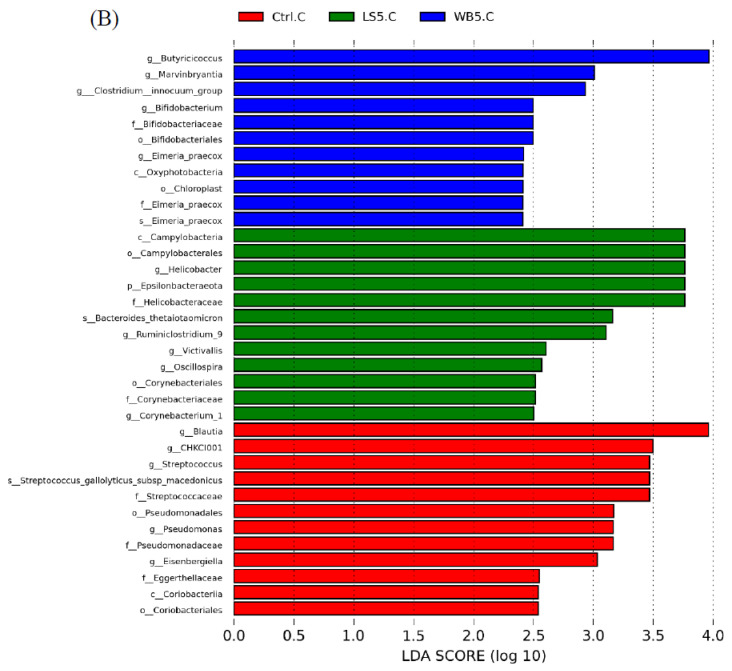
LefSE showing the microbiota composition in the ileum (**A**) and cecum (**B**) of 35-day-old broilers. Ctrl: control group; WB5: broilers supplemented with 5% wheat bran; LS5: broilers supplemented with 5% *Laetiporus sulphureus* wheat bran. Results are means of six samples obtained from the individual birds of each of the control and experimental groups.

LEfSE results showed that, in ileum, the order Lactobacillales, class Bacilli, and species *L. salivarius* had a higher proportion in the 5% WB group. The proportion of the family Lactobacillaceae, genera *Lactobacillus*, *Ruminococcus*, *Desulfovibrio*, *Pediococcus*, *Ordoribacter*, and *Intestinomonas* in the ileum were higher in the 5% LS group (Figure 5A). In the control group, the relative abundance of phylum Epsilonbacteraeota, class Campylobacteria, order Campylobacterales, family Bacillaceae, Helicobacteraceae, Muribaculaceae, Carnobacteriaceae, and genera *Bacillus*, *Helicobacter*, *Jeotgalibaca*, *Blautia*, *Eisenbergiella*, and *Barnesiella* was higher than in other groups (Figure 5A). The diagram of LEfSE shows that cecum Oxyphotobacteria at the class level, Bifidobacteriaceae at the family level, Bifidobacteriales and Chloroplast at the order level, *Butyricicoccus, Bifidobacterium*, *Marvinbryantia*, the *Clostridium innocuum* group, and *Eimeria praecox* at the genus level, and *Eimeria praecox* at the species level were more abundant than other groups (Figure 5B). In the 5% LS group, the composition of Epsilonbacteraeota at the phylum level, Campylobacteria at the class level, Campylobacterales and Corynebacteriales at the order level, Helicobacteraceae and Corynebacteriaceae at the family level, *Helicobacter*, *Victivallis*, *Oscillospira*, and *Ruminiclostridium* at the genus level, and *Bacteroides thetaiotaomicron* at the species level were more numerous than in the other groups (Figure 5B). For the control group, Coriobacteriia at the class level, Pseudomonadales and Coriobacteriales at the order level, Streptococcaceae, Pseudomonadaceae, and Eggerthellaceae at the family level, *Streptococcus*, *Pseudomonas*, *Eisenbergiella*, and *Blautia* at the genus level, and *Streptococcus gallolyticus* subsp. *macedonicus* were higher than in other groups (Figure 5B).

### 3.7. Effects of LS on the KEGG Pathway in the Ileum and Cecum of the Broilers

**Figure 6:**
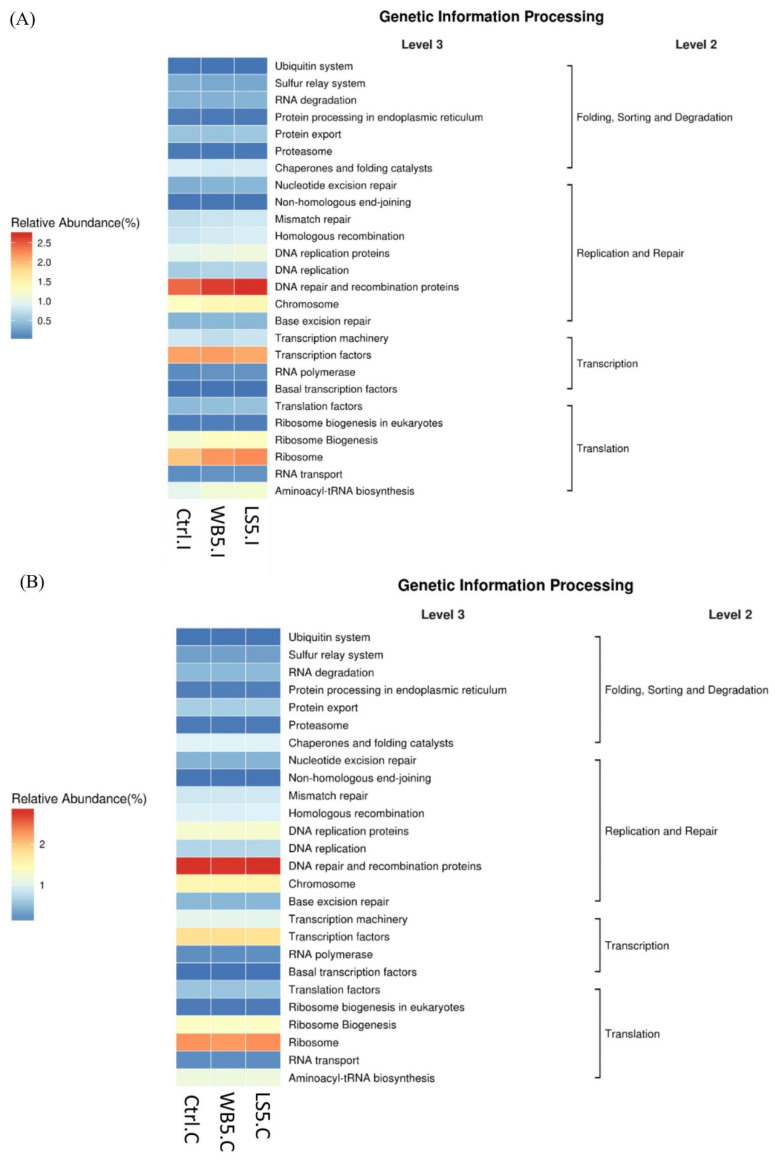
Effects of LS on the KEGG pathway in the ileum (**A**) and cecum (**B**) of broilers. Ctrl: control group; WB5: broilers supplemented with 5% wheat bran; LS5: broilers supplemented with 5% *Laetiporus sulphureus* wheat bran. Results are means of six samples obtained from the individual birds of each of the control and experimental groups.

According to the function prediction on ileum microbiota on the genetic information process level (Level 3), DNA repair and the recombination proteins on the replication and protein category and ribosomes on the translation category had a stronger function in the 5% LS supplementation compared to other groups (Figure 6A). Function predict on cecum microbiota showed similar trends between each group (Figure 6B).

### 3.8. Effects of LS on the Total VFA, pH Value, and Ammonium-Nitrogen of the Ileal and Cecal Digesta in the Broilers

The effects of LS on selected indexes of ileal and cecal digesta are displayed in Table 3. Results showed that 5% WB and 5% LS supplementation significantly elevated the total VFA concentration in cecum compared to the control group, while total VFA concentration in the ileum was not significantly different across each group. The pH values were significantly lower in the ileum and cecum in the 5% LS group, compared to the 5% WB and control groups. In addition, the 5% LS supplementation group showed significantly lower levels of ammonium-nitrogen in the ileum and cecum compared to the control and 5% WB-supplemented groups.

### 3.9. Effects of LS on the Lactate Concentration of Ieal and Cecal Digesta in Boilers

The lactate concentration in the ileum and cecum digesta of the LS- and WB-supplemented broilers are demonstrated in Table 4. Ileum digesta of the 5% LS-supplemented group had significantly higher levels of lactate. Furthermore, cecal digesta also showed that the lactate concentration in the 5% LS supplemented group was significantly higher compared to the other groups.

## 4. Discussion

Due to the important role of intestinal microbiota for host digestion, immunity, and growth performance, it is crucial to investigate how LS manipulates the intestinal microbiota in broilers and also the correlations on the physical and biological performance affected by the supplementation of LS. A diversity of intestinal microbiota in broilers is largely influenced by feed composition. Furthermore, the bacterial diversity within the intestinal tract is positively correlated with a better FCR [26], and a low diversity could induce the overgrowing of certain pathogenic bacteria and lead to the development of disease [27]. In our study, a 5% WB and 5% LS supplementation showed no effects on the alpha diversity of the ileum and cecum of the broilers. This was different from most of the studies that reported high-fiber and NSPs included in diets promote the diversity of intestinal microbiota [28]. However, Wanzenböck et al. [29] reported that 15% supplementation of WB and *Pleurotus eryngii*-fermented WB had no effects on the alpha diversity of the jejunum, ileum, and cecum in laying hens; that finding is close to our results. It is possible that the supplementation level of either LS or WB in this study was not enough to affect the alpha diversity in the intestine of broiler chickens.

Intestinal microbiota in chicken is dominated by *Lactobacillus* spp., *Enterococcus* spp., and various Clostridiaceae [30]. The ileum contains butyrate-producing bacteria, which might influence the absorption and utilization of nutrients and affect the performance of the broilers. The cecum is the organ that contained the greatest diversity and abundance of microbiota taxonomy because of the fermentation process that occurs as a result of its longer retention time (12 to 20 h). The cecum is dominated by *Lactobacillus* species, *Ruminococcus*, *Clostridium*, *Eubacterium*, and *Faecalibacterium*, which are linked to cellulose and polysaccharides-rich feedstuffs that had resistance to digestion enzymes in the foregut [1]. In our study, it was highlighted that the population of Lactobacillaceae in both the ileum and cecum was promoted by both LS and WB supplementation, which might be due to the effect of the NSPs contained in LS and WB, which acted as prebiotics to modulate the microbiota towards beneficial bacteria. However, the mean difference comparison results revealed that the LS group was more enriched in *Lactobacillus*, compared to WB group; this could be attributed to the exopolysaccharides, structural polysaccharides, xylanase, and phenolic compounds of *L. sulphureus* produced during the fermentation process (examined in our previous study) [20]. Some studies had reported the effects of fungal fermentation products on increasing the *Lactobacillus* population in broilers [6,10,11]. *Lactobacillus* are a group of commensal bacteria known to modulate the immune function of the intestine and promote the health of the host. *Lactobacillus* have been reported to release peptides with low molecular weight that induce immune activation [31]. Furthermore, by producing a wide variety of short-chain fatty acids (SCFAs), *Lactobacillus* can inhibit some pathogenic bacterial species either directly or by lowering the intestinal pH [32]. Lai et al. [10] demonstrated that co-fermented *Pleurotus eryngii* stalk residues and soybean hulls by *Aureobasidium pullulans* improved cecal lactic acid bacteria, compared to the control group, and claimed that the polysaccharide content in the fermentation product had beneficial effects on improving the intestinal microbiota. Nowak et al. [33] reported that polysaccharides of Polish wild mushroom had effects on promoting the growth of *Lactobacillus* strains in an in vitro test. *Ganoderma lucidum* polysaccharides was also reported to increase the relative abundance of Lactobacillales in the intestine of mice that suffered from chronic pancreatitis [13]. Polysaccharides are suitable substrates for probiotic bacteria in the GIT. LS contained various resources of polysaccharides, including α-glucan and β-glucan [34,35]; these glucans had been proved to have prebiotic effects on intestinal Lactobacilli, which increased its population [36,37]. Furthermore, polysaccharides from several wild mushroom species, including *Cantharellus cibarius*, *Amanita muscaria*, *Fomes fomentarius*, *Ganoderma applanatum*, *Morchella conica*, and *Xerocomus badius*, also exhibited similar growth-promoting effects on *Lactibacillus* strains [34,38]. Furthermore, xylanase has been reported to improve Lactobacilli and *Bifidobacteria* counts in the caecum of broilers fed a corn/soy-based diet [28]; the study’s findings are in congruence with this study, as it indicates that LS also exerted xylanase activity. The reason the LS-produced xylanase has increases the population of *Lactobacillus* in the GIT of the broilers might be because the xylanase could produce xylooligosaccharides from xylan (a type of NSP that is abundant in WB) that acts as prebiotics [39,40,41]. Furthermore, according to the gene coding, xylosidase of GH3 and GH43 are present in the *Lactobacillus* genomes and GH43 is characterized by its high efficiency in utilizing xylobiose, xylotriose, and xylotetraose, which reasoned the promoted population of the *Lactobacillus* strains in the LS-supplemented group [42,43]. Phenolic compounds, which show antimicrobial effects, can also potentially promote the growth of beneficial bacteria [44]. For example, Barroso et al. [45] used batch culture fermentation of feces and reported that red wine extracts contained phenolic compounds that could promote the growth of *Lacotbacillus* spp. and *Ruminococcus* spp. On the other hand, Viveros et al. [46] supplied broiler chickens with grape pomace concentrate and grape seed extracts that served as the source of phenolic compounds and demonstrated that populations of *Entrococcus* spp. and *Lactobacillus* spp. were increased. Furthermore, several reports had proved that phenolic compounds can be utilize by intestinal bacteria and helped the biotransformation of phenolic acids and increase its bioavailability [47,48]. All of this evidence suggest that the phenomenon and mechanism of LS supplementation could improve the growth of *Lactobacillus* in the intestines of broilers.

Studies have linked the growth performance of chickens with the structure of their intestinal microbiota and the population of certain commensal bacteria due to their important role in the absorption and utilization of nutrients [49,50,51]. In our study, LS increased the population of *Lactobacillus*, *Tenericutes*, and *Faecalibacterium* in the ileum, and it has been reported that these bacteria are correlated with a higher body weight in broilers [49,50]. The increased population of *Lactobacillus* in ceca is positively correlated with the enhancement of body weight in birds, while Lachnospiraceae and Ruminococcaceae, both SCFA producers that aid the digestion of dietary fiber, are related to a better FCR [51]. This intestinal microbiota profile, positively correlated to growth performances, was common in the broilers supplemented with 5% LS, which showed a better FCR and EBI during 35 d. Interestingly, pathogenic bacteria had a higher relative population in the WB-supplemented group, such as ileal *Streptococcus* and cecal *Shigella*, which suggested that a 5% WB inclusion in the broilers’ diet might promote some unfavored microbiota composition that were reported to potentially retard the growth performance of broilers [52]. With reference to the results, the 5% WB supplementation had no adverse effects on the growth performance of the broilers.

The cecum and ileum of the 5% LS supplementation group had lower Proteobacteria and higher Firmicutes at the phylum level. The phylum Proteobacteria include many pathogens that correlate with the production of pro-inflammatory cytokines, linked to low productivity in broilers [49,50]. Firmicutes promotes anti-inflammatory states, and the promotion of the genus *Faecalibacterium* was also shown to improve the anti-inflammatory status [1,53]. Furthermore, the increment in the genus *Lactobacillus* and for the Ruminococcaceae were linked to the production of short-chain fatty acids (SCFA), which were reported to inactivate the NF-κB pathway and inhibit the production of pro-inflammatory cytokines in dendric cells and macrophages [54]. These results correlate to our previous study that demonstrated the anti-inflammatory effects of LS by dampening the secretion of pro-inflammatory cytokines, such as IL-1β and TNF-α [20].

To understand the effects of LS on the microbiota structure of broilers, we further investigated the contents in ileal and cecal digesta to validate that the change in gut microbiota had effects on the environment of the intestine. According to our results, the increment in SCFA-producing bacteria (*Lactobacillus* and Ruminococcaceae) in the intestine should result in an increment in the detected VFA of the LS group, when compared to the control group. However, the total VFA was not significantly increased; there was only a slight elevation in the cecum, which could be investigated more precisely by applying the gas chromatography method to determine the individual content of the SCFAs, such as acetate, butyrate, propionate, etc. The increment in the lactate concentration and the decrement in the pH value were evidence of increased *Lactobacillus* that can acidify the GIT environment. A similar result was reported by Lin et al. [6] who observed an elevation in the lactic acid in the cecum of broilers accompanied by an improved population of lactic acid bacteria in chickens supplemented with *Trichoderma pseudokoningii*-fermented enzyme powder. Ammonium-nitrogen was produced by the incomplete utilization of excessive proteins in animal feed, which could cause environmental pollution and odor problems. Furthermore, Proteobacteria were reported to be increased in population in diseases related to active protein fermentation in the GIT, such as inflammatory bowel disease [55]. The decreased ammonium-nitrogen detected in the ileum and cecum of the LS-supplemented broilers could be the result of a reduction in the Proteobacteria population, which also explained the protein-fermentation activities in the ileum and cecum. NSP-degrading enzymes can also facilitate the production of oligosaccharides that are preferred by the bacteria and prevent the fermentation of proteins that cause putrefaction in the cecum [5]. In our study, xylanase contained in the LS helped reduce the ammonium-nitrogen since carbohydrase was reported to increase the concentration of organic acids, reduce ammonia production, and increase the SCFA concentration. This indicates the hydrolysis fragmentation of the NSPs by beneficial intestinal bacteria [56]. The evidence provided in this section suggests that the change in microbiota transformed the intestinal environments of broilers and could potentially favor the growth and health of broilers.

## 5. Conclusions

The present study provides some valuable investigations for developing LS as a potential functional feed ingredient. A 5% LS supplementation is able to improve the growth performance of the broiler chickens, which was dependent to the modulation of the microbiota composition and the improvement of the environment in the ileum and cecum.

## Figures and Tables

**Table 1:** Effects of the LS supplementation on the growth performance of 35-day-old broilers ^1^.

Items	Experimental Diets			SEM	*p*-Value
	Control	5% WB	5% LS		
FBW (g)	2393.94	2407.64	2499.46	17.12	0.11
ADG (g/chick/d)	67.05	67.44	69.65	0.50	0.25
FCR (kg feed/kg gain)	1.47 ^b^	1.45 ^b^	1.41 ^a^	0.01	0.04
Viability (%)	93.75	91.25	93.75	0.72	0.31
EBI ^2^	424.28 ^b^	413.89 ^b^	468.72 ^a^	4.21	0.02

^1^ The results are provided as the means of four replicates (20 birds/replicate) in each control and treatment group. ^2^ EBI = (ADG (g) × viability %)/FCR × 10. FBW: final body weight; ADG: average daily weight gain; FCR: feed conversion ratio; EBI: European broiler index; WB: wheat bran; LS: *Laetiporus sulphureus*-fermented wheat bran; SEM: standard error of the mean. ^a,b^ Means within the same rows without the same superscript letter are significantly different (*p* < 0.05).

**Table 2:** Diversity indices of the ileal and cecal microbiota of 35-day-old broilers ^1^.

Items	Experimental Diets			SEM	*p*-Value
	Control	5% WB	5% LS		
Ileum					
Ace	139.66	212.56	232.67	16.93	0.09
Chao1	138.29	208.83	238.80	18.03	0.10
Simpson	0.70	0.68	0.78	0.03	0.44
Shannon	3.08	2.32	3.15	0.30	0.47
Cecum					
Ace	487.91	463.67	478.53	6.55	0.34
Chao1	497.03	467.15	484.96	7.74	0.31
Simpson	0.97	0.96	0.95	0.01	0.59
Shannon	6.34	6.02	6.14	0.12	0.53

^1^ Results are means of six samples obtained from the individual birds of each of the control and experimental groups. WB: wheat bran; LS: *Laetiporus sulphureus*-fermented wheat bran; SEM: standard error of the mean.

**Table 3:** Effects of LS supplementation on the total VFA concentrations, pH values, and NH_3_-N of the intestinal content of 35-day-old broilers ^1^.

Items	Experimental Diets			SEM	*p*-Values
	Control	5% WB	5% LS		
Total VFA	μmole/g				
Ileum	11.25	11.73	11.36	0.09	0.03
Cecum	32.74 ^b^	34.01 ^a^	35.20 ^a^	0.34	0.02
NH_3_-N					
Ileum	151.32 ^a^	152.27 ^a^	140.24 ^b^	0.0004	1.02
Cecum	190.02 ^a^	189.60 ^a^	174.57 ^b^	0.011	1.74
pH value					
Ileum	5.54 ^a^	5.55 ^a^	5.01 ^b^	0.07	0.03
Cecum	7.25 ^b^	7.40 ^a^	7.08 ^c^	0.02	0.004

^1^ Results are means of six samples obtained from the individual birds of each of the control and experimental groups. WB: wheat bran; LS: *Laetiporus sulphureus*-fermented wheat bran; SEM: standard error of the mean. ^a,b,c^ Means within the same rows without the same superscript letter are significantly different (*p* < 0.05).

**Table 4:** Effects of LS supplementation on the lactate concentration of the intestinal content of 35-day-old broilers ^1^.

Items	Experimental Diets			SEM	*p*-Values
	Control	5% WB	5% LS		
	μM				
Ileum	43.16 ^b^	43.82 ^b^	58.02 ^a^	0.87	0.002
Cecum	90.88 ^b^	92.73 ^b^	108.12 ^a^	1.07	0.0003

^1^ Results are means of six samples obtained from the individual birds of each of the control and experimental groups. WB: wheat bran; LS: *Laetiporus sulphureus* fermented wheat bran; SEM: standard error of the mean. ^a,b^ Means within the same rows without the same superscript letter are significantly different (*p* < 0.05).

## Supplementary Materials

The following are available online at https://www.mdpi.com/2076-2615/10/9/1457/s1, Table S1: Ingredients and chemical composition of the experimental diets for broilers.

## Abbreviations

LS	*Laetiporus sulphureus*-fermented wheat bran
WB	Wheat bran
OTU	Operational taxonomic units
VFA	Volatile fatty acid
FCR	Feed conversion ratio
EBI	European Broiler Index
NSPs	Non-starch polysaccharides
GIT	Gastrointestinal tract
BCRC	Bioresource Collection and Research Center
MEA	Malt extract agar
MEB	Malt extract broth
ADG	Average daily weight gains
HPLC	High-performance liquid chromatography
