# Supplementary material for: Effects of Laetiporus sulphureus-Fermented Wheat Bran on Growth Performance, Intestinal Microbiota and Digesta Characteristics in Broiler Chickens
Source: Animals (Basel). 2020 Aug 20;10(9):1457. doi: 10.3390/ani10091457 (PMC7552699; doi:10.3390/ani10091457)
Supplement: Supplementary file 1 [file animals-10-01457-s001.zip › Suppl. table 1.docx]

**Supplementary table 1.** Ingredients and chemical composition of the experimental diets for broilers

| Ingredients | Starter diet (1-21 days) | | | | Finisher diet (22-35 days) | | | |
| --- | --- | --- | --- | --- | --- | --- | --- | --- |
|  | Control | 5% WB | 5% LS |  | | Control | 5% WB | 5% LS |
|  | ----------------------------------------g/kg---------------------------------------------- | | | | | | | |
| Corn | 524.9 | 458.7 | 458.4 |  | | 549.5 | 482.9 | 482.9 |
| WB | 0 | 50.0 | 0 |  | | 0 | 50.0 | 0 |
| LS | 0 | 0 | 50.0 |  | | 0 | 0 | 50.0 |
| Soybean meal, CP 44% | 320.0 | 167.3 | 167.3 |  | | 16.6 | 185.6 | 185.6 |
| Fish meal, CP 60% | 50.0 | 50.0 | 50.0 |  | | 320.6 | 167.8 | 167.8 |
| Full fat soybean meal | 41.4 | 209.8 | 210.1 |  | | 30.0 | 30.0 | 30.0 |
| Soybean oil | 30.0 | 30.0 | 30.0 |  | | 10.6 | 10.6 | 10.6 |
| Limestone | 11.6 | 11.6 | 11.6 |  | | 12.2 | 12.2 | 12.2 |
| Monocalcium phosphate | 11.2 | 11.2 | 11.2 |  | | 3.4 | 3.3 | 3.3 |
| _DL_-Methionine | 3.4 | 3.7 | 3.7 |  | | 50.0 | 50.0 | 50.0 |
| Salt | 2.9 | 2.8 | 2.8 |  | | 1.3 | 1.5 | 1.5 |
| _L_-Lysine HCl | 1.8 | 2.1 | 2.1 |  | | 3.0 | 3.3 | 3.3 |
| Choline-Cl (60%) | 0.8 | 0.8 | 0.8 |  | | 0.8 | 0.8 | 0.8 |
| Vitamin premix^1^ | 1.0 | 1.0 | 1.0 |  | | 1.0 | 1.0 | 1.0 |
| Mineral premix^2^ | 1.0 | 1.0 | 1.0 |  | | 1.0 | 1.0 | 1.0 |
| Total | 1000.0 | 1000.0 | 1000.0 |  | | 1000.0 | 1000.0 | 1000.0 |
| Calculated nutrient value |  |  |  |  | |  |  |  |
| ME, kcal/kg | 3050.0 | 3050.0 | 3050.0 |  | | 3175.0 | 3175.0 | 3175.0 |
| Dry matter, % | 88.28 | 88.85 | 89.11 |  | | 88.31 | 88.88 | 89.15 |
| Crude protein, % | 23.0 | 23.0 | 23.0 |  | | 21.0 | 21.0 | 21.0 |
| Crude fat, % | 6.04 | 8.86 | 8.64 |  | | 7.56 | 10.39 | 10.17 |
| Calcium, % | 1.05 | 1.05 | 1.05 |  | | 0.90 | 0.90 | 0.90 |
| Total phosphorus, % | 0.73 | 0.73 | 0.73 |  | | 0.68 | 0.67 | 0.67 |
| Available phosphorus, % | 0.50 | 0.50 | 0.50 |  | | 0.45 | 0.45 | 0.45 |
| Lysine, % | 1.43 | 1.43 | 1.43 |  | | 1.25 | 1.25 | 1.25 |
| Methionine, % | 0.73 | 0.74 | 0.74 |  | | 0.65 | 0.66 | 0.66 |
| Cysteine, % | 0.34 | 0.32 | 0.32 |  | | 0.31 | 0.30 | 0.30 |
| Analyzed nutrient value |  |  |  |  | |  |  |  |
| Dry matter, % | 88.22 | 88.67 | 89.11 |  | | 88.71 | 88.79 | 89.04 |
| Crude protein, % DM | 23.54 | 23.48 | 23.45 |  | | 21.83 | 21.77 | 21.67 |
| Crude fat, % DM | 6.32 | 8.89 | 8.63 |  | | 7.23 | 10.33 | 10.73 |

WB: wheat bran; LS: *Laetiporus sulphureus* fermented wheat bran.

^1^Supplied per kg of diet: Vit. A 15000 U; Vit. D_3_ 3000 U; Vit. E 30 mg; Vit. K_3_ 4 mg; Riboflavin 8 mg; Pyridoxine 5 mg; Vit. B_12_ 25 μg; Ca-pantothenate 19 mg; Niacin 50 mg; Folic acid 1.5 mg; Biotin 60 μg.

^2^Supplied per kg of diet: Co (CoCO_3_) 0.255 mg; Cu (CuSO_4_･5H_2_O) 10.8 mg; Fe (FeSO­_4_･H_2_O) 90 mg; Zn (ZnO) 68.4 mg; Mn (MnSO_4_･H_2_O) 90mg; Se (Na_2_SeO_3_) 0.18 mg.
